# Survival Versus Attraction Advantages and Talent Selection in Sport

**DOI:** 10.1186/s40798-022-00409-y

**Published:** 2022-01-29

**Authors:** Joseph Baker, Kathryn Johnston, Nick Wattie

**Affiliations:** School of Kinesiology and Health Science, York University, Toronto, Canada

**Keywords:** Athlete selection, Evolutionary psychology, Talent, Athlete development

## Abstract

Athlete selection (often referred to as talent selection) reflects the end point of what is a complex decision-making process coaches, administrators, and/or scouts use when deciding who remains and who is removed from a sample of potential athletes. In this paper, we conceptualize athlete selection as an evolutionary process where selection pressures (e.g., performance demands, system limitations) influence the value of one trait/characteristic over another. Athletes are selected either through demonstrating enhanced performance (survival advantages) or by having characteristics that are desirable to the coach/recruiter making the selection (attraction advantages). Based on these varying pressures, our understanding of whether profiles of current athletes represent the actual elements of performance necessary for success or simply those most needed for selection at key points in athlete development is extremely limited.

## Key Points

- Evolutionary models can be used to better understand how sport systems behave.
- An evolutionary model of athlete selection suggests athletes succeed in development systems due to survival (i.e., performance) and attraction effects.
- In order to better understand inefficiencies in talent selection and athlete development, researchers need to determine which variables are related to survival (i.e., performance) and which result from attraction.

## Background

The data-rich context of contemporary sport makes it fertile ground for scientific exploration. One area that has been especially attractive amongst researchers from a variety of fields (e.g., sport science as well as psychology and economics) relates to the evaluation of athletes’ long-term potential. Across an athlete’s development, those wishing to access increasingly higher levels of competition typically have to successfully navigate many barriers, the most fundamental being selection to a team. Athlete selection (more commonly referred to as talent selection) reflects the end point of what is a complex decision-making process coaches, administrators, and/or scouts use when deciding who remains and who is removed from a sample of potential athletes (e.g., when coaches select who will play on the local competitive versus recreational teams or when professional teams choose players in a sports draft). At regular intervals across an athlete’s development, these selection checkpoints restrict or permit who moves forward in the system. Research in this area suggests our evaluations of ‘talent’ or ‘potential’ are generally quite poor [1], with increases in prediction duration leading to increases in prediction difficulty. [2] A range of potential mechanisms have been proposed to explain these poor levels of accuracy (e.g., cognitive biases, system inefficiencies) [3].

## An Evolutionary Approach to Athlete Selection

Contemporary evolutionary theory provides the most robust explanation of the natural world and our place in it. Furthermore, it can help us understand how complex systems with varying biological and environmental elements evolve over time. For instance, several researchers [4–6] have used evolutionary models to understand the dynamics of sport systems. Gould [5], for example, found that changes in performance in major league baseball were best explained using evolutionary models showing consistent improvement within a relatively controlled system, as evidenced through indicators of overall system performance (e.g., decreasing deviation in offensive indicators such as batting performance and winning percentage, as well as increased performance on defensive indicators such as fielding averages across the population of baseball players). Chatterjee and Yilmaz [4] noted that without accounting for a system’s evolutionary performance, one could easily make the erroneous assumption that a 0.420 batting average in 1911 is significantly better than a 0.390 average in 1980. Similarly, Puterman et al. [6] examined changes in the distribution of left- versus right-oriented players throughout the history of the National Hockey League, noting an eventual stabilization in the proportion of players from different orientations reflecting an ‘evolutionarily stable strategy’. Collectively, these studies highlight the potential value of applying evolutionary theory to understanding sport performance.

The chief mechanism driving evolution in a complex system is ‘selection pressure’, which broadly relates to the range of factors that influence the selection of one trait/characteristic over another. If an organism has a beneficial trait that is more likely to be selected, it has an advantage over its peers. In biological evolution, this can be manifested through a survival advantage, relating to a greater likelihood of survival such as found in animals with more effective defensive mechanisms, or through a reproductive advantage, which relates to a greater likelihood of finding a mate, and passing on one’s genes. Importantly, these advantages are tied to the environment in which an organism finds itself. As environmental conditions change selection pressures change.

## Survival Versus Attraction Advantages

In this paper, we argue analogous selection pressures exist in high performance sport systems, and these pressures may be useful for helping us understand the psychology of talent selection. For instance, in athlete selection contexts, athletes can be chosen by being superior performers compared to their peers, broadly relating to the notion of ‘survival’ due to superior ‘fitness’. From an evolutionary perspective, this fitness relates to the athlete’s ability to thrive and outperform in the specific setting they inhabit. We consider these as reflecting *‘survival advantages’.* An example of this process in action is seen in the ‘expanding universe of athletes’ bodies’ [7]. As sports have matured, athletes’ bodies have become more distinct, with inter-sport anthropometric variation increasing, and intra-sport variation of bodies decreasing. In short, there has been a generational move from the ‘average’ body as ideal to increasingly specialized bodies (often extremes) that better align with the performance demands of sports.^1^

A less acknowledged, but related, advantage some athletes possess that affects their likelihood of being selected relates to whether they possess desirable traits/characteristics that the coach/scout explicitly or implicitly chooses. These reflect *‘attraction advantages’,* that individual coaches/scouts may deem either implicitly or explicitly ‘attractive’ in regards to what they are looking for in a selection setting. Attraction and survival advantages are not mutually exclusive; the former usually serves to reinforce the latter. The relative age effect, for example, is a persistent selection bias found in many sports driven by coaches’ mis-selection of relatively older players over relatively younger players [8–10]. Presumably, coaches over-select relatively older players because they perceive them as having immediate performance advantages and/or greater potential for long-term success (i.e., they are more attractive). Unfortunately, coaches have misinterpreted advanced growth and maturation compared to their peers as reflecting greater potential. Factors leading to attraction advantages likely change across development due to differing demands and constraints in the high-performance athlete pathway. For instance, early in a gymnast’s development, coaches may look at parents to get rough estimates of an athlete’s eventual adult height. Later, when the population of gymnasts remaining in the system has become homogeneous for height, other factors such as ability to manage emotions under pressure or ‘coachability’ may become more important. In this example, the environmental context has changed, leading to different selection pressures.

There is undoubtedly overlap between these two types of selection pressure. Those who are objectively superior performers are likely more ‘attractive’ to coaches seeking to select athletes with the greatest potential. Attraction advantages often relate to the factors that also confer survival advantages; there is likely a co-evolution of these advantages. This phenomenon is common in nature, arguably best evidenced by the peacock’s tail. The tail appears to have no survival benefit, and in fact it seems to make the male a greater target for predators; however, a larger and more elaborate tail (as reflected in the number of ‘eyes’) is believed to be a sign of superior fitness (i.e., potential for survival), which results in being seen as more attractive by peahens. Understanding the selection pressures related to athlete assessment at different stages of development would help uncover the mechanisms at work. For instance, do these different selection mechanisms change the strength of the effect on selection decisions (e.g., is attraction more important than survival earlier in the pathway)? Indeed, many inaccurate athlete selection decisions may result from misguided or overgeneralized attraction effects; the equivalent of judging the size and quality of a peacock’s tail before the tail has finished growing (see below).

## What Do We Really Know About Athlete Selection?

Ideally, athlete selection would reflect the end result of explicit choices regarding what characteristics are best suited for long-term success. Unfortunately, in sport settings, it is not possible to know the accuracy of deciding to choose one athlete over another since their careers diverge after this selection is made. One athlete moves forward into a system where they compete against better competition, with more capable peers and access to superior resources, while the other is often left outside this system, either by the system explicitly denying access or implicitly by the athlete integrating this information into their motivation and competency beliefs. Moreover, early attraction effects almost certainly lead to improved survival effects, such as when a player who is ‘likeable’ (an attraction effect) is given more opportunities to develop than someone ‘less likeable’ and eventually develops into a superior player due to these skill development opportunities. Due to these design limitations, the ultimate value of qualities such as coachability [12] and/or anti-social personality characteristics [13] as predictors of future success is unknown. Over time, these traits will be over-selected for, demonstrating strong consistency and robustness thereby giving the impression they are important for long term development when in fact they are only important for ‘selection’. Dawkins’ [14] concept of ‘selfish genes’ is based on the notion that the qualities selected for in a competitive environment are not necessarily the most beneficial for the long-term success of the species, but rather are the most desirable genes/traits at that given moment in time. Similarly, our understanding of whether profiles of current athletes represent the actual elements of performance necessary for success or simply those most needed for selection is extremely limited.

The interplay between survival and attraction advantages may help us to better understand the secular trends and challenges of talent identification and development. The specific constraints [15] associated with performance in varying sports, and resultant sport-specific athlete characteristics [7], likely reflect a kind of ‘evolutionary environment of adaptedness’^2^ (EEA). That is, athletes’ characteristics (e.g., anthropometrics) and performances reflect the environments in which those elements provide advantages. However, these constraints change and evolve over time. Additionally, the elements related to performance demands in the elite, adult context are fundamentally different (and possibly diametrically opposed) to those at lower levels of development. Although this has yet to be confirmed empirically, given the changes noted in youth sport over the past few decades, we hypothesize that the attraction advantages resulting from and associated with the EEA at the highest levels of sport, now pervade youth sport. This overgeneralization and/or extension of the attraction advantages related to elite, adult sport to youth contexts is at least partially responsible for the biases and inaccuracies (Type I and II errors) associated with early talent identification. Alternatively, youth selectors who are more concerned with immediate short-term performance than with long-term potential, will simply be selecting characteristics that fit an EEA for developmental sport. Because the survival and attraction advantages of youth and elite adult sport reflect the same characteristics to some degree,^3^ albeit on different scales, the systems’ two EEA (developmental and elite) can and do produce expert athletes. However, like judging a peacock’s tail before it is fully grown, conferring attraction advantages to youth athletes increases the cost of misestimating the survival advantages of an athlete.

To be sure, these effects will be difficult to test experimentally, at least using traditional approaches. The time frames associated with athlete development and the range of potential variables affecting development make designs like randomized controlled trials prohibitive or unfeasible. However, if we focus on the convergence of evidence in an area, produced through research synthesis methods like meta-analyses as well as systematic and scoping reviews, answers may be possible. For instance, if we consider the research evidence on the value of personality characteristics for athletic success, certain facets of personality (e.g., conscientiousness) are related to team and individual performance [16]. However, it is not possible to determine the extent to which these qualities were important for long-term success or just important at one point in time for being retained in the athlete development system. Moreover, without appropriate control groups and longitudinal designs, it will be impossible to tell. However, it may be possible to compare across sports having more or less rigid development structures, to see if these same qualities are equally valued across contexts. The notion here is that the qualities necessary for eventual success should be relatively stable across similar domains.

Importantly, the distinction between attraction and survival effects emphasizes the importance of establishing a strong theoretical rationale for the relationships under investigation. For instance, how is the personality characteristic of conscientiousness linked with performance? Is it because those who are conscientious are more obvious in how they engage and attend during training and therefore are more likely to be noticed by their coach as being ‘coachable’ and/or is being conscientious directly related to greater success?

Using evolutionary models to shift how we conceptualize athlete selection would involve a change in how we think about cause and effect. Evolutionary psychology, for instance, makes the distinction between ‘proximate’ or ‘immediate’ causes (e.g., an athlete was selected because they were seen as ‘coachable’) and ‘ultimate’ causes (e.g., ‘coachability’ is more likely to be chosen in selection settings because the athlete-coach relationship often requires a close connection and coaches may be drawn to athletes who are perceived to be ‘coachable’).[17] Both of these examples deal with the same characteristic (i.e., coachability) but position it in different ways. While both are important for understanding this process and how it can be effectively managed by practitioners, research in this area has largely neglected ultimate causes for proximal ones.

## Conclusion

In summary, we argue for a more nuanced perspective on the psychology of athlete selection that recognizes inefficiencies in the system may be driven by incongruence between survival and attraction effects, and the dynamic nature of performance environments. Considering the limitations in how we select athletes, particularly at younger ages, developing a more comprehensive understanding of what these selections mean for their long-term development will only come from an understanding of both proximate and ultimate causes.

## Data Availability

Not applicable.

## Footnotes

1 Importantly, change can occur over long periods of time, and sometimes over relatively short periods of time (e.g., structural changes identified in finch’s beaks within only a couple years [11]). The same is likely true in sport.

2 The environment in which organisms are best adapted to ‘survive’ in.

3 The notion and practice of performance benchmarks as predictive tools underpins this notion.
